# The Australian Trauma Registry (ATR): a leading clinical quality registry

**DOI:** 10.1007/s00068-023-02288-8

**Published:** 2023-06-22

**Authors:** Angela Fischer, Mark Fitzgerald, Kate Curtis, Zsolt J. Balogh

**Affiliations:** 1 Department of Traumatology, John Hunter Hospital, Newcastle, NSW Australia; 2 Injury and Trauma Research Program, Hunter Medical Research Institute, Newcastle, NSW Australia; 3 Discipline of Surgery, School of Medicine and Public Health, University of Newcastle, Newcastle, NSW Australia; 4 Department of Surgery, Central Clinical School, Trauma Services, The Alfred, National Trauma Research Institute, Monash University, Melbourne, VIC Australia; 5 Susan Wakil School of Nursing and Midwifery, Faculty of Medicine and Health, University of Sydney, Sydney, Australia; 6 Illawarra Shoalhaven Local Health District, Shoalhaven, NSW Australia

**Keywords:** Trauma, Polytrauma, Trauma registry, Trauma quality improvement, Trauma systems

## Abstract

Operating since 2012 under the auspices of the Australian Trauma Quality Improvement Program (AusTQIP), the Australian Trauma Registry (ATR) has established itself as a leading clinical quality registry (CQR). Initially developed as a national program for improved safety and quality trauma care across Australian trauma centers, it has since expanded to include New Zealand, becoming one of the few bi-national trauma registries. The registry has recorded close to 100,000 episodes of care for severely injured patients since its inception, with 10.7% growth in annual inclusions. The ATR, administered by the National Trauma Research Institute (NTRI), monitors the continuum of trauma care from pre-hospital settings, to discharge from definitive care. Collection and analysis of data about severely injured trauma patients, their injuries, management and outcomes, aims to inform future improvements to health service provision and reduce preventable morbidity and mortality.

## Introduction

Trauma continues to be a significant contributor to the burden of disease in Australia, with injury the leading cause of hospitalization and death for people aged 1–44, accounting for 7.6% of Australia’s health expenditure ($8.9 billion), in the 2020–2021 financial year [1]. The collection and analysis of injured patient data through detailed trauma registries, is essential to quantifying the extent of injury, and to capture the impact and economic burden of injury. Registries have proven critical in facilitating comparison of management and benchmarking across institutions, monitoring patterns of injury and informing injury prevention strategies, and associated management [2]. International consensus that the capacity to audit is fundamental in the management and improvement of any trauma system and drives change, is arguably the main impetus for funding of the current Australasian Trauma Registry (ATR) [3]. The value of this registry has been recognized in a publication from the Australian Commission on Safety and Quality in Healthcare (ACSQHS), on the prioritization of registries [4, 5].

## History of trauma care in Australia

Australia is a geographically large but sparsely populated country, with a land mass equivalent to the United State of America and a current population of over 26 million [6, 7]. Whilst the distribution of the population is predominantly along the east and southeastern seaboard, with a smaller concentration in the southwest corner, there are many communities in remote and rural regions with large distances between medical facilities, and varying levels of medical care available [8, 9].

Historically, trauma care has been led by emergency departments with surgical and other specialty support. This has evolved over the last 30 years since injury was first recognized as a national health priority in Australia in 1986 [6, 10]. Increased trauma volume, complexity, population growth and the development of contemporary trauma management strategies, called for the consolidation of comprehensive trauma care [6, 10].

Insufficient standardization and error in trauma management has contributed significantly to preventable or potentially preventable morbidity and mortality [11]. Most of these errors were a result of the correct diagnostic or therapeutic measures are not performed in the right measure, at the right time, or in the right order [11, 12].

The Early Management of Severe Trauma (EMST) guidelines were introduced in 1988, adapted under license from the Advanced Trauma Life Support (ATLS) guidelines of the American College of Surgeons by The Royal Australasian College of Surgeons (RACS) [13]. This has ensured a standardized approach to managing trauma for all emergency and trauma clinicians across the nation. In addition, organized trauma systems, first introduced in Australia in 1992 have also been established to improve trauma patient outcomes [13]. Trauma systems across the nation are at varying stages of evolution, with multiple factors influencing the development of trauma systems in each region [8].

Designated trauma centers are situated in metropolitan areas; therefore, many Australian trauma patients are assessed, stabilized, admitted or treated in non-trauma centers. For example, in the state of New South Wales, approximately 29% of seriously injured patients are initially managed outside a major (Level-1) trauma center each year [14]. Those who suffer major trauma in rural Australia are twice as likely to die than their metropolitan counterparts [15, 16].

## Current Australian trauma system

Each of Australia’s 6 states and 2 territories has jurisdictional control over the management of its own public health system. Medical care is delivered by a combination of public and private providers; however, the requirement for integrated multi-disciplinary care and clinical acuity dictates that major trauma is definitively managed within the public health system [6]. Private facilities in Australia contribute significantly to care of minor orthopedic trauma and provision of rehabilitation services [6].

A complex network of patient care has evolved to manage major trauma, with each of the states and territories having its own bespoke structure of trauma hubs. These are public hospitals designated as either Major Trauma Service (MTS; Level-1 equivalent) or Regional Trauma Service (RTS; Level-3 equivalent); with a protocolized retrieval system to ensure timely access to appropriate services for those who are injured in rural and regional areas [6]. The function of the MTS in its geographical region is co-ordination of care, and support of peripheral hospitals with provision of expert clinical advice, trauma education, and development of trauma policies and guidelines.

Patients who meet definitional criteria for major trauma are transported to the highest-level trauma service, within the individual state’s designated timeframe [17]. Regional Trauma Services have 24/7 emergency departments, and intensive care units with consistent general surgical and orthopedic cover; hence able to resuscitate and stabilize patients ahead of transfer to an MTS [18–20]. Rural hospitals receiving trauma patients whose needs exceed the capabilities of that hospital, and travel time exceeds the prescribed timeframe to the nearest RTS or MTS, initiate resuscitation whilst awaiting retrieval by specialist medical teams. Retrieval service providers may be tasked to the scene or to retrieve from peripheral hospitals, whilst providing expert clinical advice to support rural or regional clinicians in caring for the complex trauma patient [6].

Most states provide subspecialized units for transfer of patients with burns or spinal cord injury, outside the usual geographically based referral patterns. Pediatric trauma is also managed in designated Children’s Hospitals, which may or may-not be co-located with adult MTS [6]. Despite their separate reporting structures, there is some integration of trauma networks across state borders; particularly where geography lends itself to shorter transit times [6]. Additionally, a national response plan exists for mass casualty incidents, which may challenge any single trauma system beyond its capabilities, in the form of an integrated response across the trauma networks of bordering states [21].

Each Australian state has a governing body to monitor the trauma system, comprised of working groups or committees, supported by governance structures and key stakeholders working to deliver trauma initiatives. Their role is to oversee, co-ordinate and support the state's trauma system, using data from the state trauma registry to inform process improvement. Performance is assessed against agreed quality improvement and performance frameworks with preventable and potentially preventable mortality or other concerning deficiencies in care reported to and investigated by trauma services, local health districts and on occasion the Ministry of Health [22–27].

A leading mechanism for quality improvement and trauma endorsement in Australia and New Zealand is the RACS Trauma Care Verification Program. Introduced around 2 decades ago, this multi-disciplinary, inter-collegiate benchmarking process aims to assist hospitals to analyze their trauma systems of care, identifying strengths, weaknesses and critical deficiencies of the hospital’s trauma service; with recommendations and evidence in the detailed report used to support a business case for additional resource necessary for optimal care of injured patients in accordance with international standards [27–30]. Studies have demonstrated verification-driven changes have the ability to impact hospital expenditure, length of stay and reduce trauma mortality [28–31]; with the focus of Trauma Care Verification recently shifted from individual trauma centers to entire trauma systems [32].

The Australian and New Zealand Association for the Surgery of Trauma (ANZAST) in 2019 [33] developed the Post-Fellowship Education and Training (PFET) Program in Trauma Surgery administered by the General Surgeons Australia (GSA) Trauma Training Committee (TTC) [34]. The TTC includes vascular, orthopedic and general surgeons, from Australia and New Zealand, as well as military and trainee representatives [34]. The development of strong relations between the American Association for the Surgery of Trauma (AAST) and ANZAST has helped to foster collaboration across the two regions; with The Journal of Trauma and Acute Care Surgery the official journal of ANZAST since its inception [35].

To complement the specialized skills training offered by ANZAST, The Master of Traumatology offered by the University of Newcastle has been developed in response to increasing demand for trauma specialized health care professionals. It is Australia’s only online post-graduate trauma program, and one of only two post-graduate programs worldwide [36]. The program aims to provide an advanced educational foundation for those wishing to specialize in trauma management and improve candidature for further vocational training. Graduates will build their career with contemporary knowledge and skills in trauma management and the function of trauma systems internationally [36].

## The Australian Trauma Registry (ATR)

In 1993, the need for a national trauma registry in Australia was initially tabled by the National Road Trauma Advisory Council, supported by RACS and the Australasian Trauma Society (ATS) [37, 38]. This early advocacy laid the foundation for the formation a decade later of the Australian and New Zealand National Trauma Registry Consortium (NTRC). Majority of funding to the consortium from its inception in 2003 was supplied by the commonwealth, and subsequently supported by New South Wales Institute of Trauma Injury Management (ITIM), the ATS and RACS [37–39]. The championship of these bodies enabled the NTRC to commit to the task of standardizing trauma data collection to enable benchmarking, and develop a trauma minimum dataset (MDS) that would serve both Australia and New Zealand. The dataset is now known as the Bi-National Trauma Minimum Dataset (BNTMDS) [37–39].

Whilst the development of the BNTMDS was in progress, the NTRC ceased operations; however, decades of commitment and support to an agreed MDS, laid a firm foundation for the next phase of development—the Australian Trauma Quality Improvement Program and the ATR (AusTQIP-ATR) [37, 38]. AusTQIP including the ATR began operations in 2012 with representation from stakeholders in all states and territories. A survey to determine the status of quality improvement and data collection capacity at all of the major trauma centers (MTCs) around the country was undertaken, identifying information gaps with the major trauma centers, and resulting in a formalized Collaboration Agreement in May 2014, with executive endorsement from the organizations that would contribute data to the ATR [3, 37, 38]. The ATR is currently funded by The National Trauma Research Institute, the Australian Federal Department of Health, The Australian Federal Bureau of Infrastructure and Transport Research Economics and the New Zealand Accident Compensation Cooperation, with in-kind funding received from Monash University.

The ATR currently collects pre-hospital and inpatient data from 28 Australian and 7 New Zealand Level-1 Major Trauma Centers (MTCs), on the most severely injured trauma patients; defined as an Injury Severity Score (ISS) greater than 12 (based on AIS 2005 Update 2008), or in-hospital death following injury [40]. Exclusion criteria include delayed admissions greater than seven days following injury, drowning, hanging, ingestion/poisoning not resulting in injury, foreign bodies without injury, iatrogenic injuries, isolated neck of femur fracture, isolated pathological injury, geriatric mortality following superficial injury only (contusions, abrasions, or lacerations) and/or have coexisting morbidity precipitating injury or death (e.g., stroke, malignancy, heart failure, advanced frailty by Rockwood Clinical Frailty Score, etc.) [40].

Data are prospectively collected by trauma clinicians or data managers certified in American Association for the Surgery of trauma Abbreviated Injury Scale (AAST AIS) coding, with sound knowledge of the requirements of the ATR. It is then collated quarterly according to the Bi-National Minimum Trauma Dataset (BNMTDS); with data elements mapped from existing hospital and state-based registries, according to standard definitions. Data elements not collected by existing registries are otherwise not obtained by the ATR [39]. The dataset includes but is not limited to demographic data, details of the injury event, pre-hospital observations/transport/timing, referral facilities, critical care admission and ventilation days, injuries sustained, treatments received, access to computed tomography and critical procedures, in-hospital observations, bloodwork, complications, co-morbidities and discharge status/destination [40, 41]. The BNMTDS currently records 90 datapoints. The ATR continues to recruit sites, capturing population-based data of severely injured trauma patients [40].

Data submitted to the ATR are subject to various validity checks prior to data processing. These include correct classification per ICD-10-AM and AIS 2005 (Updated 2008) codes and date and time formats and chronology. If unable to pass validation, an error file is generated and notification sent to the submitting site to address and correct the error, if possible [40]. Contributions vary widely as some facilities do not have all the BNTMDS data points readily available. However, this continues to improve as updated data systems and improved data quality processes are implemented [40].

Severely injured trauma patient episodes of 11,254 were collected by the ATR during the 2020–21 financial year [40], excluding New Zealand data, the 28 major trauma centers across Australia provided 9,413 episodes Transport-related injuries and falls remain the leading cause of in-hospital admissions, accounting for 84.2 per cent of all severe injuries bi-nationally [14]. The epidemiology of severe injuries in recent years appears to be changing, however, with an increase in predominantly older patients severely injured from low falls, resulting in greater morbidity and mortality [38]. In 2020–21, the ATR reports low falls as accounting for 45.9 per cent of all severely injured, 65 years or above. High falls also contribute significantly to morbidity and mortality in this age group, with household hazards such as stairs and ladders identified as a target for injury prevention programs. Low fall mortality in this age group (24.3%) was well above the overall bi-national all-cause mortality rate of 9.3 per cent [14]. On average, 29% of severely injured patients were transferred versus direct admissions to a major trauma service with median time from injury to hospital for direct admissions, greater than 90 min. Therefore, majority of life saving interventions performed in the first hour or two, occur prior to arrival at the MTS. Variability in the delivery of interventions across jurisdictions calls for careful comparison and refinement to optimize outcomes [14]. Majority of patients remain in ED greater than 5 h, despite the National Emergency Access Target (NEAT) of 4 h. Inpatient rehabilitation disposition upon discharge varies between 5 and 26% across services, with major changes in configuration of services and more in-home programs [14]. A full list of publications, and a collection of annual reports/collaborator reports are available for public access on the ATR website [42, 43]. The ATR is also in the process of developing an interactive dashboard for existing sites, to allow better access and enable clinicians to visualize and manipulate data to make meaningful comparisons with other sites and juristictions.

The Australian Trauma Quality Improvement Program (AusTQIP) Steering Committee established the ATR to be a repository of trauma related data voluntarily reported by contributing trauma centers. The ATR is governed by the ATR Board and AusTQIP Steering Committee, with all proposals for ATR data use to be submitted to the ATR Manager and subject to approval by the ATR Board [40, 44]. Access to New Zealand data requires additional written approval from the Data Governance Group of the NZ Trauma Registry. Once the data request has been reviewed and approved by the ATR Board, this request will subsequently be made by the ATR Manager [44].

The ATR database is stored within a secure system by the data host organization, Monash University. Data may only be accessed by researchers via the ATR Portal (for sites that contribute data to the ATR) or the secure online research platform (SeRP) for non-contributing sites/organizations. SeRP provides researchers the ability to access data via secure remote access [44]. The ATR Data Manager, as custodian, will have access to SeRP, as will the Data Host Director and any pre-authorized researchers [44]. Affiliate appointments are provided for external researchers, with access provided once the project has been approved [41].

## Obstacles and possibilities of the registry

A key activity of the ATR is to gather and disseminate data that will improve trauma care. These data are accessible to all contributors, clinical researchers, government, and the public (subject to ethical and governance policies), and is widely encouraged. However, facilities encounter many barriers to complete and accurate data collation. These include insufficient funding and resource for data collection, as a variety of sources require interrogation (i.e., pre-hospital case sheets, patient’s medical records, radiology reports, and lab databases, etc.) [45]. This results in significant duplication of data, which could in the future be negated through an interface between registry software and clinical systems.

A substantial proportion of injury burden is currently not represented by the ATR. Data are exclusively collected on hospital admissions, and a large proportion of both major and minor trauma patients are not treated at trauma centers contributing data to the ATR. This inhibits monitoring of those injured and managed in rural and regional locations [3]. The ATR continues the effort to recruit and expand the number of contributing sites; with an additional 10 sites added to the 2022 report, increasing rural and regional respresentation. Scene fatalities, patients dead on arrival and those not meeting “major trauma” criteria are also excluded; therefore, coronial and emergency services data could also be considered for inclusion to ensure data completeness across the spectrum of care [3].

Clinicians have indicated a need to capture additional patient outcome data such as quality of life and long-term functional outcomes, and acknowledged the benefit of a possible interface with other databases to track these outcomes [46, 47].

## Comparison to other registries

A number of ATR fields lack comparability with international datasets throughout Europe, USA and Canada; limiting utility of benchmarking to international standards. 27 of the 90 BNMDS elements are comparable to the 35 data elements of the Utstein Template. Considerable international collaboration would be required to standardize worldwide trauma data; and it appears there is no greater comparability between the international datasets reviewed during the creation of the BNTMDS, than between any one of these datasets and the BNTMDS [40].

## Examples of recent research activities/quality improvement activities using registry data

The pivotal role of trauma registries in providing high quality data for quality improvement is well understood; however, the ATR is not yet in a position to be able to achieve this, as reliable monitoring and valid measurement of processes and clinical outcomes rely on near-complete inclusion of all eligible patients. However, AusTQIP provides a nationally coordinated and integrated approach to systems quality improvement and patient safety, and continue to report on patterns of serious injury on the basis of understanding the annual burden of trauma [40]. With increased facility and ATR resources, and continued expansion of contributing sites, the potential for the ATR to affect change and positive outcomes is certainly possible.

## Future prospects/perspectives

It is envisaged that as ATR data completeness and coverage improves, the data will increasingly be used to monitoring the effect of public health initiatives, including road safety campaigns and other community issues such as the growing incidence of ladder falls in elderly males [3, 38]. Risk-adjusted modeling will facilitate benchmarking of clinical processes and outcomes between jurisdictions, that will inform trauma improvements. Other planned developments include better use of available IT systems, and a targeted MDS to capture presentations to Emergency Departments as a result of road trauma [38].

## Conclusions

The vision for an Australian national trauma registry has long been championed by a community of committed trauma advocates. The long and arduous process, culminating in secured commonwealth funding and the ongoing financial support of ATS and RACS, resulted in the highly anticipated establishment of the ATR. The ATR is now well placed to monitor and impact the quality of care received by the severely injured; with a systems-based approach, backed by high quality aggregate data, trauma experts, policy makers and legislators essential in reducing the consequence and burden of traumatic injury.
